# Evaluation of gene amplification and protein expression of HER-2/neu in esophageal squamous cell carcinoma using Fluorescence *in situ *Hybridization (FISH) and immunohistochemistry

**DOI:** 10.1186/1471-2407-9-6

**Published:** 2009-01-07

**Authors:** Yukie Sato-Kuwabara, José I Neves, José HTG Fregnani, Rubens A Sallum, Fernando A Soares

**Affiliations:** 1 Department of Anatomic Pathology, Hospital AC Camargo, Rua Antônio Prudente, 211, 1o. andar, São Paulo, SP, CEP: 01509-010, Brazil; 2 Department of Morphology, School of Medical Sciences of Santa Casa of São Paulo, Rua Dr. Cesário Motta Jr., 61, São Paulo, SP, CEP: 01221-020, Brazil; 3 Department of Abdominal Surgery, Hospital AC Camargo, Rua Antônio Prudente, 211, 1o. andar, São Paulo, SP, CEP: 01509-010, Brazil

## Abstract

**Background:**

Esophageal squamous cell carcinoma (ESCC) is the sixth most frequent neoplasia in Brazil. It is usually associated with a poor prognosis because it is often at an advanced stage when diagnosed and there is a high frequency of lymph node metastases. It is important to know what prognostic factors can facilitate diagnosis, optimize therapeutic decisions, and improve the survival of these patients. A member of the epidermal growth factor receptor (EGFR) family, c-erbB-2, has received much attention because of its therapeutic implications; however, few studies involving fluorescence *in situ *hybridization (FISH) analysis of HER-2/neu gene amplification and protein expression in ESCC have been conducted. The aim of this study was to verify the presence of HER-2/neu gene amplification using FISH, and to correlate the results with immunohistochemical expression and clinical-pathological findings.

**Methods:**

One hundred and ninety-nine ESCC cases were evaluated using the Tissue Microarray (TMA) technique. A polyclonal antibody against c-erbB-2 was used for immunohistochemistry. Analyses were based on the membrane staining pattern. The results were classified according to the Herceptest criteria (DAKO): negative (0/1+), potential positive (2+) and positive (3+). The FISH reactions were performed according to the FISH HER2 PharmDx (DAKO) protocol. In each case, 100 tumor nuclei were evaluated. Cases showing a gene/CEN17 fluorescence ratio ≥ 2 were considered positive for gene amplification.

**Results:**

The c-erbB-2 expression was negative in 117/185 cases (63.2%) and positive in 68 (36.8%), of which 56 (30.3%) were 2+ and 12 (6.5%) were 3+. No significant associations were found among protein expression, clinicopathological data and overall survival. Among the 47 cases analyzed, 38 (80.9%) showed no gene amplification while 9 (19.1%) showed amplification, as demonstrated by FISH. Cases that were negative (0/1+) and potential positive (2+) for c-erbB-2 expression by immunohistochemistry showed no gene amplification. However, all cases with gene amplification were positive (3+) by immunohistochemistry. According to univariate analysis, there was a significant difference (p = 0.003) in survival rates when cases with and without HER-2/neu amplification were compared.

**Conclusion:**

Our data demonstrate the correspondence between gene amplification and protein expression of HER-2/neu. Gene amplification is an indicator of poor prognosis in ESCC.

## Background

Squamous cell carcinoma is the most frequent histological type of cancer of the esophagus (ESCC) [1]. It occurs predominantly in developing countries and is the sixth most common neoplasia in Brazil [2]. ESCC generally has a poor prognosis because it is usually in an advanced stage at the time of diagnosis. Its epidemiological characteristics suggest the involvement of environmental factors in its etiology [3-5].

The mortality rate is still high despite the development of many therapeutic modalities in addition to surgical resection. Esophagectomy with lymph node dissection has significant morbidity and mortality rates [5-7]. Therefore, it is important to understand the prognostic factors that determine the most appropriate surgical approach in order to select patients for target-directed therapies.

The family of epidermal growth factor receptors comprises four homologous molecules: EGFR, c-erbB-2, c-erbB-3 and c-erbB-4. Some reports have suggested that abnormal activation of kinase activity in these receptors has an important role in tumor development and progression in ESCC [8,9]. Activation of these receptors induces cell migration and aggregation as well as hyperproliferation and epithelial cell differentiation [1]. The importance of these receptors has been studied in many malignant neoplasms, but mainly in breast carcinoma, leading to specific treatments to inactivate them [10].

Gene amplification has been described in many tumor types and is associated with poor prognosis [11]. When identified, gene amplification is an important indicator for more intensive treatment, mainly in breast and lung cancer [12-15]. It is analyzed by fluorescence (FISH) or chromogenic (CISH) in situ hybridization to measure the gene copy number.

Studies have verified that the HER-2/neu (c-erbB-2) oncogene (located on chromosome 17) is amplified and overexpressed in many tumor types, and have compared these results with immunohistochemical expression and clinicopathological data using Tissue Microarray [16-20] or conventional slides [21-23]. Amplification and overexpression of HER-2/neu (c-erbB-2) predict an especially poor prognosis in breast cancer.

Few studies have used FISH to evaluate HER-2/neu amplification in ESCC; these reports show low gene amplification frequencies in this tumor [24,25]. Mimura *et al*. (2005) found that 11% of cases showed HER-2/neu amplification, including all cases considered 3+ by immunohistochemical analysis and 3/6 (50%) of those considered 2+ [24]. These authors also demonstrated that cases with gene amplification had lower survival rates. A study by Sunpaweravong *et al*. (2005) showed gene amplification in 2% of cases, but this factor was not associated with overall survival [26]. Similar results were presented by Reichelt *et al*. (2007), in which 5% of the ESCC were positive for amplification of HER-2/neu; however, amplification did not influence prognosis [25].

Although recognition of gene amplification may be a key factor in therapeutic decision-making, there is a paucity of data regarding HER-2/neu amplification in ESCC. In this study, we detect gene amplification of HER-2/neu by fluorescence *in situ *hybridization and evaluate protein expression by immunohistochemistry in ESCC. We discuss the relationship between the clinicopathological features of this disease and the prognostic value of HER-2/neu amplification.

## Methods

### ESSC cases and specimens

This study was performed with the approval of the Ethics Committee of Hospital A. C. Camargo, São Paulo, Brazil (Protocol n. 530/03). It utilized archival formalin-fixed paraffin-embedded tissues from surgical specimens of 199 ESCC cases treated at Hospital A. C. Camargo between 1980 and 1999. From these 199 cases, 427 samples were analyzed using the Tissue Microarray (TMA) technique.

The samples were distributed into three TMA paraffin blocks containing 1 mm cores of 143 samples in each of two blocks and 141 in a third. One hundred and fifty-three cases were represented by more than one sample in the TMA blocks. All samples were spotted in duplicate, corresponding to different areas of the same original paraffin block. Slides of 3 μm were obtained by adhesives specific for the technique (Instrumedics Inc.). The TMA blocks were constructed with "Manual Tissue Arrayer I" (Beecher Instruments Inc.).

For all cases we reviewed age, gender, ethnicity, location of primary tumor, histological grade, extent of infiltration, lymphatic and venous invasion, and evolution of disease. The clinicopathological data are summarized in Table 1.

**Table 1:** Clinicopathological findings of ESCC cases selected for this study

Variable	Category	N (%)	Total
**Gender**	**Male**	161 (80.9)	199
	**Female**	38 (19.1)	

**Ethnicity**	**Caucasian**	148 (74.4)	199
	**Not Caucasian**	42 (21.1)	
	**No data available**	9 (4.5)	

**Tumor location**	**Upper**	34 (17.1)	199
	**Middle**	104 (52.3)	
	**Lower**	50 (25.1)	
	**No data available**	11 (5.5)	

**Histological grade**	**G1**	62 (31.2)	199
	**G2**	93 (46.7)	
	**G3**	42 (21.1)	
	**G4**	2 (1)	

**Infiltration level**	**Basal layer**	9 (4.5)	199
	**Muscular**	42 (21.1)	
	**Adventitia**	148 (74.4)	

**Lymphatic invasion**	**Not observed**	160 (80.4)	199
	**Present**	39 (19.6)	

**Venous invasion**	**Not observed**	58 (29.1)	199
	**Present**	141 (70.9)	

**pN**	**Negative**	107 (53.8)	199
	**Positive**	89 (44.7)	
	**No data available**	3 (1.5)	

**Surgical margins**	**Not committed**	6 (3.0)	199
	**Committed**	188 (94.5)	
	**No data available**	5 (2.5)	

**Stage**	**I**	6 (3)	199
	**II**	86 (43.2)	
	**III**	53 (26.6)	
	**IV**	52 (26.1)	
	**No data available**	2 (1)	

**Tumor recurrence**	**Negative**	87 (43.7)	199
	**Positive**	110 (55.3)	
	**No data available**	2 (1)	

**Evolution of disease**	**Death from disease**	153 (76,9)	199
	**Alive without disease**	7 (3.5)	
	**Alive with disease**	1 (0.5)	
	**No follow-up**	38 (19.1)	

The follow-up time ranged from 1 to 200 months with an average of 21.8 months. The ages of the 199 patients selected for this study ranged from 37 to 80 years, with a mean of 57 years (standard deviation = 9).

### Immunohistochemistry

The immunohistochemical reactions were realized by the streptavidin-biotin-peroxidase complex technique (StreptABC, DAKO, Denmark). The tissue sections were deparaffinized and incubated in citrate buffer in a pressure cooker for antigenic retrieval, then endogenous peroxidase activity was blocked with 3% H_2_0_2_. The sections were then incubated with polyclonal primary antibodies against c-erbB-2 (1:500, A0485, DAKO, Denmark). Subsequently, they were incubated in secondary biotinylated antibody from LSAB+ peroxidase Kit (DAKO, K0690, Denmark), followed by incubation with Streptavidin HRP (DAKO, Denmark), and were counterstained with Hematoxylin.

Immunohistochemical analyses of c-erbB-2 expression describe the intensity and staining pattern of tumor cells. The FDA-recognized test, the Herceptest™ (DAKO), describes four categories: no staining, or weak staining in fewer than 10% of the tumor cells (0); weak staining in part of the membrane in more than 10% of the tumor cells (1+); complete staining of the membrane with weak or moderate intensity in more than 10% of the neoplastic cells (2+); and strong staining in more than 10% (3+).

Each sample was categorized in one of three groups according to the following criteria: most of the cores had 0 and/or 1+ expression (Negative Group); at least 50% of the cores had 2+ expression and none had 3+ (Potential Positive Group); and at least one case had 3+ expression and none had 0/1+ (Positive Group).

Two slides of each TMA block, separated by about 40 sections, were subjected to immunohistochemistry. Because each sample was spotted in duplicate in each TMA block, there were four immunohistochemical analyses for each specimen. In addition, because each case could be represented by more than one sample in the TMA blocks, more than four cores could be obtained in each case for each marker.

### Dual-color Fluorescence *in situ *Hybridization

One slide of each TMA block was subjected to hybridization reactions. HER2/neu amplification was analyzed using FISH HER2 PharmDx (Dako, K5331, Denmark), which contains both fluorescently-labeled HER2/neu gene and chromosome 17 centromere probes.

In brief, the sections were incubated at 56°C overnight and deparaffinized by washing in xylene, ethanol and distilled water. After incubation in 0.2 M HCl at room temperature for 20 minutes they were heat-pretreated in citrate buffer (2 × SSC, pH 6.0) at 80°C for 1 to 1.5 hours. They were then digested with pepsin at room temperature for 8–14 minutes, rinsed in 2 × SSC at room temperature for 2 minutes and dehydrated in graded ethanol (75, 80, and 100%) for 2 minutes.

After the HER2/CEN17 probe mix was applied to the dry slides, the tissue area was coverslipped and sealed with rubber cement. The slides were then incubated in hybridizer (Hybridizer Instrument for *in situ *hybridization, DAKO, S2450, Denmark) for denaturation at 82°C for 5 minutes and hybridization at 45°C for about 18 hours.

Posthybridization washes were performed in urea/0.1 × SSX at 45°C for 30 minutes and in 2 × SSC at room temperature for 2 minutes. The slides were dehydrated in graded ethanol, and after application of 15 μL of mounting medium containing 4',6'-diamidino-2-phenylindole (DAPI), the tissue area was coverslipped.

FISH analyses were performed according to the HER2 FISH PharmDx (Dako, Denmark) criteria. In each case, 100 non-overlapped, intact interphase tumor nuclei identified by DAPI staining were evaluated, and gene (red signal) and CEN17 (green signal) copy numbers in each nucleus were assessed. The cases were considered to be amplified when the average copy number ratio, HER2/CEN17, was ≥ 2.0 in all nuclei evaluated or when the HER2 signals formed a tight gene cluster. Among the cases in which the gene was not amplified, samples showing more than four copies of the HER2 gene and more than four CEN17 in more than 10% of the tumor cells were considered to be polysomic for chromosome 17.

### Statistical analyses

All statistical analyses were performed using SPSS for Windows 13.0, SPSS Inc. Categorical variables were compared by the Pearson chi-square test or Fisher's exact test, depending on the expected values found in the contingency table. The overall survival rates were calculated using the Kaplan-Meier method and the curves were compared by the log-rank test. In all statistical tests, the alpha error was set at 5%. The survival period was calculated from the date of hospital admission to death or the date of last follow-up.

## Results

### c-erbB-2 immunohistochemistry

**Figure 1:**
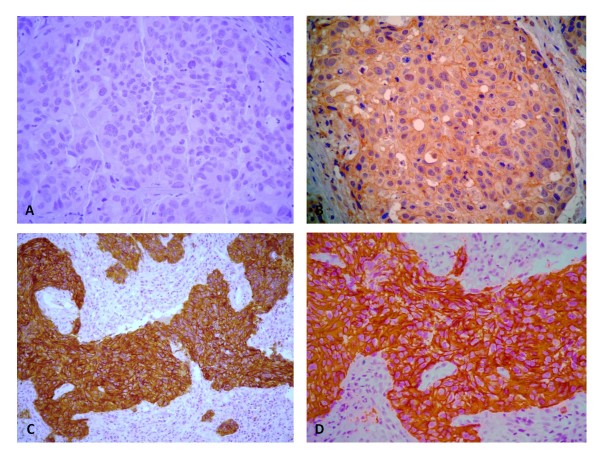
**Immunohistochemistry showing c-erbB-2 membrane staining in ESCC**. (a) sample negative for expression of this protein, 400×; (b) positive case 2+, 400×; (c) and (d) positive cases 3+, 400×.

Expression of c-erbB-2 was analyzed in 185 cases of ESCC according to the membrane staining pattern of the tumor cells. One hundred and seventeen cases (63.2%) were negative and 68 (36.8%) were positive, of which 56 (30.3%) were 2+ and 12 (6.5%) were 3+ (Figure 1).

**Figure 2:**
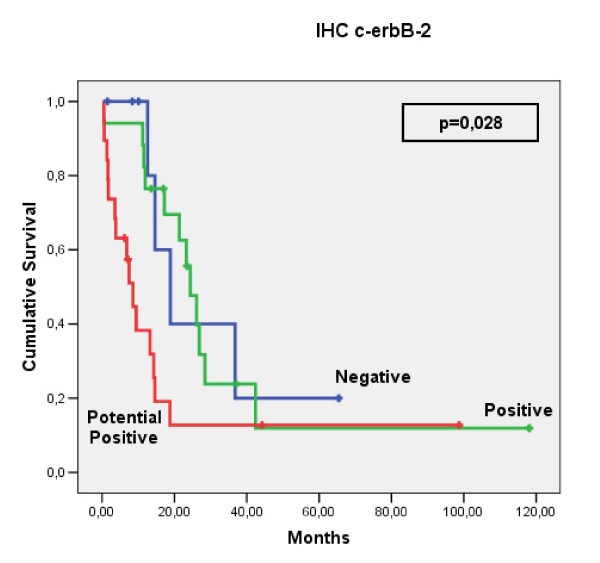
**Overall survival according to HER-2/neu expression**. c-erbB-2 expression in 185 cases of ESCC.

Statistical analysis revealed no significant association between c-erbB-2 expression and the clinicopathological findings. The data are summarized in Table 2. Furthermore, univariate analysis showed that c-erbB-2 expression was not significantly associated with overall survival (Figure 2).

**Table 2:** Association between c-erbB-2 expression and clinicopathological data in 185 cases of ESCC analyzed by TMA

Variables	Category	c-erbB-2			
		
		Negative [0 and 1+] (%)	Positive [2 and 3+] (%)	Total	p value
**Gender**	**Male**	97 (82.9)	52 (76.5)	149	0.337
	**Female**	20 (17.1)	16 (23.5)	36	

**Age**	**< 60 years**	61 (54.5)	46 (69.7)	107	0.057
	≥ **60 years**	51 (45.5)	20 (30.3)	71	

**Ethnicity**	**Caucasian**	84 (77.1)	52 (77.6)	136	1.000
	**No Caucasian**	25 (22.9)	15 (22.4)	40	

**Tumor location**	**Upper**	18 (16.2)	12 (18.8)	30	0.781
	**Middle**	65 (58.6)	34 (53.1)	99	
	**Lower**	28 (25.2)	18 (28.1)	46	

**Histological grade**	**G1+G2**	87 (74.4)	57 (83.8)	144	0.147
	**G3+G4**	30 (25.6)	11 (16.2)	41	

**Infiltration level**	**Basal layer**	5 (4.3)	0 (0)	5	NA
	**Muscular**	22 (18.8)	17 (25)	39	
	**Adventitia**	90 (76.9)	51 (75)	141	

**Lymphatic invasion**	**Not observed**	30 (25.6)	19 (27.9)	49	0.733
	**Present**	87 (74.4)	49 (72.1)	136	

**Venous invasion**	**Not observed**	95 (81.2)	54 (79.4)	149	0.848
	**Present**	22 (18.8)	14 (20.6)	36	

**pN**	**Negative**	63 (53.8)	36 (52.9)	99	1.000
	**Positive**	54 (46.2)	32 (47.1)	86	

**Surgical margins**	**Not committed**	110 (95.7)	67 (98.5)	177	NA
	**Committed**	5 (4.3)	1 (1.5)	6	

**Stage**	**1+2**	58 (49.6)	27 (39.7)	85	0.222
	**3+4**	59 (50.4)	41 (60.3)	100	

**Recurrence**	**Negative**	49 (42.6)	28 (41.8)	77	1.000
	**Positive**	66 (57.4)	39 (58.2)	105	

NA: not available

### HER-2/neu amplification

**Figure 3:**
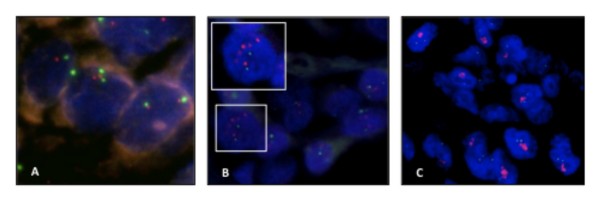
**Analysis of HER-2/neu gene amplification in 47 cases of ESCC**. (a) Samples with no amplification; (b) case considered amplified. In detail, nucleus shows ratio ≥ 2 and (c) gene amplification showing cluster pattern. 1000×.

The same cases evaluated by immunohistochemistry were also examined using FISH. Of the 199 cases, 47 were suitable for evaluating gene amplification. In the remaining 138 cases there was no HER2 gene or CEN17 hybridization signal, or these samples showed collapsed nuclei. Gene amplification was found in nine (19.1%) of the cases; 38 (80.9%) showed no amplification (Figure 3). Among the cases in which amplification was not found, five (13.2%) had polysomy of chromosome 17.

When the FISH results were compared with the c-erbB-2 immunohistochemical data, six (66.7%) of the nine cases with gene amplification showed positive expression (3+ IHC) for this protein. Three (33.3%) were not available for immunohistochemistry, as shown in Table 3. None of the amplified cases was negative or had 2+ protein expression as assessed by immunohistochemistry. Otherwise, among the cases in which gene amplification was not found, 11 (28.9%) and 15 (39.5%) were 0/1+ and 2+ by IHC, respectively. However, six (15.8%) cases lacking gene amplification showed positive (3+ IHC) expression. Of the five cases showing polysomy of chromosome 17, two (40%) were 3+ IHC, two (40%) were 2+, and one (20%) was negative (0/1+ IHC).

**Table 3:** Comparison of gene amplification analysis and immunohistochemical results of HER-2/neu in 47 cases of ESCC

IHC c-erbB-2	FISH HER-2			
	
	Not Amplified (%)	Polysomy 17 (%)	Amplified (%)	Total
**0/1+**	10 (30.3)	1 (20)	0 (0)	11

**2+**	13 (39.4)	2 (40)	0 (0)	15

**3+**	4 (12.1)	2 (40)	6 (66.7)	12

**NA**	6 (18.2)	0 (0)	3 (33.3)	9

**Total**	33 (100)	5 (100)	9 (100)	47

NA: not available

In view of the low number of cases studied, it was not possible to compare the FISH results and clinicopathological findings statistically (data not shown).

**Figure 4:**
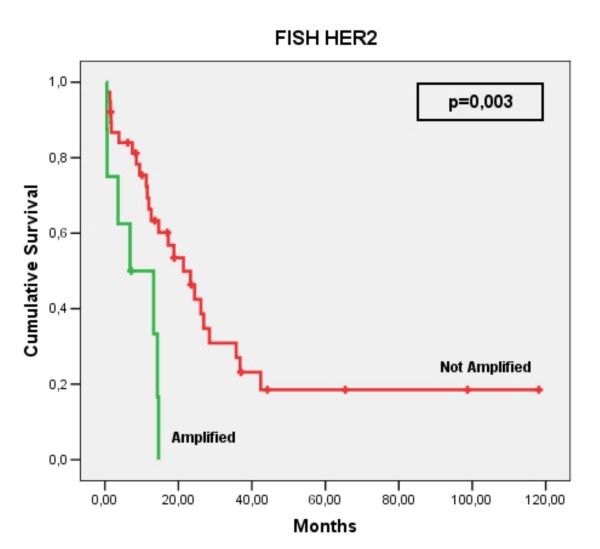
**Overall survival according to HER-2/neu amplification**. HER-2/neu amplification in 47 cases of ESCC.

According to the Kaplan-Meier method, there was a significant difference (p = 0.003) between the survival rates when cases with and without gene amplification were compared. Cases demonstrating gene amplification showed the worst survival rates (Figure 4).

## Discussion

The TMA technique provides a high-throughput means of evaluating genetic alterations in a great number of tumor samples. Sections from TMA blocks can be used for FISH, allowing gene amplification to be evaluated in hundreds of tumor samples on a single slide.

Immunohistochemical analysis showed that 36.8% of the cases were positive for expression of c-erbB-2. The few studies reporting c-erbB-2 expression in ESCC show discrepant frequencies ranging from 0 to 64% [27-31]. This variability may have resulted from differences in immunohistochemical protocols, different antibody sources used by the different authors, or different criteria for evaluating expression.

We found no significant association between c-erbB-2 expression and the clinicopathological findings. Most reported studies agree with our results and report no influence of c-erbB-2 expression on ESSC prognosis [27,28,32]; however, a study performed by Lam *et al*. (1998) demonstrated a significant association between this protein and histological grade [29], and a recent study showed that c-erbB-2 overexpression is associated with lower rates of survival [33].

The reported frequencies of HER-2/neu amplification in ESCC vary from 2 to 11% of cases [24-26]. This variability may have resulted from differences in tissue preparation, probes, and the methods used to evaluate the alterations. Our study demonstrated gene amplification in 20% of cases, higher than any previously reported frequency [24-26].

In this study, 199 cases were subjected to immunohistochemical evaluation; 47 were also suitable for FISH analysis. The remaining cases had complete loss of tumor cores from the TMA or fewer than 100 tumor nuclei were available for evaluation, or the samples were under- or over-digested, resulting in absence of hybridization signals and loss of cell and nuclear morphology.

One of the problems with the TMA technique for FISH is that fixatives such as formalin preserve the tissue structure by forming cross-bridges, which reduce the access of the probes to their targets. Another important challenge in FISH analysis is to standardize the tissue digestion on TMA: the hundreds of samples on one slide may differ in tissue type, fixation methods and age. Successful FISH analysis depends on a pretreatment protocol that is consistent in the use of digesting agents (sodium thiocyanate, pepsin or proteinase K) as well as appropriate conditions (concentration, temperature and time of incubation) to unmask the target DNA and optimize hybridization. The presence of hundreds of tissue cores on a TMA slide compounds the difficulty of optimization; a given set of conditions appropriate for some samples may over- or under-digest others.

Among the 47 cases suitable for FISH analysis, only those that were 3+ positive for immunohistochemistry showed HER-2/neu amplification (66.7%). The remaining 33.3% of the amplified cases were not suitable for immunohistochemical evaluation because there were no tumor cells in the cores or the tissue cores were completely lost from the TMA.

Mimura *et al*. (2005) found results similar to those of the present study, but as well as all the 3+ cases, 50% of their 2+ cases also showed gene amplification [24]. Another study reported a significant association between gene amplification and protein overexpression in 70% of 3+ and in 30% of 2+ amplified cases [25]. In contrast to these results, Sunpaweravong *et al*. (2005) found no significant association between gene amplification and immunohistochemical expression; the one case positive for amplification did not overexpress c-erbB-2 [26].

With respect to overall survival, our data are in agreement with the findings of Mimura *et al*. (2005), showing significant differences in survival rates in cases with gene amplification [24]. The significant association between HER-2/neu amplification and lower survival rate indicates a role for this gene alteration analysis in the prognosis of ESCC cases.

Our results did not show that c-erbB-2 expression has a prognostic value in ESCC. In contrast, Mimura *et al*. (2005) found that the survival rate in the HER-2-positive group was significantly worse than that in the HER-2-negative group. Despite the concordance between the FISH and immunohistochemistry results, only the presence of gene amplification seems to affect prognosis in ESCC. Unlike the previous report, FISH and immunohistochemistry analyses were performed on different numbers of cases (47 and 185, respectively) in the current study, which could have contributed to the discrepancy between the two studies [24]. Further studies with more cases are necessary for a better understanding of the influence of this gene on ESCC progression.

Tumors showing c-erbB-2 overexpression but not gene amplification could occur because of transcriptional or post-transcriptional alterations, polysomy of chromosome 17 or even technical problems such as the high sensitivity of the immunohistochemical procedure. In the current study, the assessment of the number of copies of chromosome 17 showed that some 3+ cases could have resulted from polysomy 17. Transcriptional or post-transcriptional alterations were not evaluated in the present study; further studies employing other techniques such as RT-PCR would be required. Alternatively, the greater sensitivity of immunohistochemistry compared with FISH could also account for this result.

Nonetheless, our results corroborate previous reports that most c-erbB-2 overexpression is caused by gene amplification [14]. There is a consensus that c-erbB-2 expression must be evaluated initially by immunohistochemistry and, if the results are not conclusive, FISH should be performed. Such a practice has been standard procedure to assist in making therapeutic decisions in breast and lung cancer cases [12-15].

Trastuzumab (Herceptin) prolongs survival in metastatic breast cancer patients whose tumours overexpress the HER2/neu protein [34]. Similarly, patients with ESCC having HER-2/neu gene amplification might also benefit from treatment with Trastuzumab, since HER-2/neu amplification indicates a group of cases in which this type of treatment could improve the prognosis [24,35].

## Conclusion

Our results indicate that HER-2/neu gene amplification is an important prognostic indicator in ESCC. Further studies with more cases and including additional techniques are necessary to verify other molecular alterations involved in tumor progression, thus assisting in the development of new therapies for ESCC.

## Competing interests

The authors declare that they have no competing interests.

## Authors' contributions

YSK designed the study, evaluated the immunohistochemistry and FISH results and wrote the manuscript. JIN carried out all the immunohistochemistry and FISH reactions. JHTGF performed all the statistical analyses. RAS performed the surgical procedures and evaluated the clinical records. FAS designed the study, assisted in analysis of immunohistochemistry results, and wrote the manuscript. All authors read and approved the manuscript.

## Pre-publication history

The pre-publication history for this paper can be accessed here:

http://www.biomedcentral.com/1471-2407/9/6/prepub
